# Modulation of Fronto-Striatal Functional Connectivity Using Transcranial Magnetic Stimulation

**DOI:** 10.3389/fnhum.2019.00190

**Published:** 2019-06-13

**Authors:** Isabel Alkhasli, Katrin Sakreida, Felix M. Mottaghy, Ferdinand Binkofski

**Affiliations:** ^1^Section Clinical Cognitive Sciences, Department of Neurology, Faculty of Medicine, RWTH Aachen University, Aachen, Germany; ^2^Department of Neurosurgery, Faculty of Medicine, RWTH Aachen University, Aachen, Germany; ^3^Department of Nuclear Medicine, Faculty of Medicine, RWTH Aachen University, Aachen, Germany; ^4^Department of Radiology and Nuclear Medicine, Maastricht University Medical Center (MUMC+), Maastricht, Netherlands; ^5^Juelich Aachen Research Alliance (JARA)—BRAIN, Juelich, Germany; ^6^Research Centre Juelich, Institute of Neuroscience and Medicine (INM-4), Juelich, Germany

**Keywords:** functional connectivity, prefrontal cortex, DLPFC, striatum, intermittent theta burst stimulation (iTBS), resting state, fronto-striatal network

## Abstract

**Background**: The fronto-striatal network is involved in various motor, cognitive, and emotional processes, such as spatial attention, working memory, decision-making, and emotion regulation. Intermittent theta burst transcranial magnetic stimulation (iTBS) has been shown to modulate functional connectivity of brain networks. Long stimulation intervals, as well as high stimulation intensities are typically applied in transcranial magnetic stimulation (TMS) therapy for mood disorders. The role of stimulation intensity on network function and homeostasis has not been explored systematically yet.

**Objective**: In this pilot study, we aimed to modulate fronto-striatal connectivity by applying iTBS at different intensities to the left dorso-lateral prefrontal cortex (DLPFC). We measured individual and group changes by comparing resting state functional magnetic resonance imaging (rsfMRI) both pre-iTBS and post-iTBS. Differential effects of individual sub- vs. supra-resting motor-threshold stimulation intensities were assessed.

**Methods**: Sixteen healthy subjects underwent excitatory iTBS at two intensities [90% and 120% of individual resting motor threshold (rMT)] on separate days. Six-hundred pulses (2 s trains, 8 s pauses, duration of 3 min, 20 s) were applied over the left DLPFC. Directly before and 7 min after stimulation, task-free rsfMRI sessions, lasting 10 min each, were conducted. Individual seed-to-seed functional connectivity changes were calculated for 10 fronto-striatal and amygdala regions of interest with the SPM toolbox DPABI.

**Results**: Sub-threshold-iTBS increased functional connectivity directly between the left DLPFC and the left and right caudate, respectively. Supra-threshold stimulation did not change fronto-striatal functional connectivity but increased functional connectivity between the right amygdala and the right caudate.

**Conclusion**: A short iTBS protocol applied at sub-threshold intensities was not only sufficient, but favorable, in order to increase bilateral fronto-striatal functional connectivity, while minimizing side effects. The absence of an increase in functional connectivity after supra-threshold stimulation was possibly caused by network homeostatic effects.

## Introduction

The fronto-striatal network is known to play a significant role in various motor, cognitive, and emotional processes (Breitenstein et al., 1998; Arnsten, 2009; Beste et al., 2012). The striatum, which is comprised of caudate, putamen, and the nucleus accumbens, receives afferents from the substantia nigra and the ventral tegmentum in the midbrain, and in turn projects to the pallidum, thalamus, globus pallidus, and substantia nigra. Additionally, there are strong output connections to the amygdala, hypothalamus, and pedunculopontine nucleus (Robbins and Everitt, 1992). Animal studies have suggested a direct modulation mechanism of the prefrontal cortex (PFC) through its projections onto the striatal neurons (Bouyer et al., 1984; Sesack and Pickel, 1992; Murase et al., 1993; Karreman and Moghaddam, 1996; Keck et al., 2002; Kanno et al., 2003, 2004). More recently, neuroimaging studies done on human subjects identified strong anatomical and functional connections between the dorsolateral PFC (DLPFC) and the dorsal-posterior caudate, while the ventrolateral PFC was found to be mainly interconnected with the ventral caudate (Leh et al., 2007; Di Martino et al., 2008; Choi et al., 2012). Functionally, the DLPFC is associated with a wide range of executive functions such as working memory, selective attention and decision making (Curtis and D’Esposito, 2003; Glenn et al., 2009) while the ventrolateral PFC is more involved in motor control (Levy and Wagner, 2011).

Resting state functional magnetic resonance imaging (rsfMRI) research is a powerful tool to reveal intrinsically, functionally connected areas by simply correlating the ongoing resting blood oxygenation level dependent (BOLD) activity of anatomically distinct areas. Areas that with synchronized ongoing activation are thought to be functionally linked. RsfMRI studies of the fronto-striatal network, as well a task-based fMRI research have, for example, been used to reveal the dysfunction of this system, which can lead to severe cognitive and behavioral, as well as emotional symptoms. Abnormal fronto-striatal network functions have been linked to neurodegenerative diseases such as Parkinson’s disease and prodromal Alzheimer dementia, as well as mood disorders and impulse-control disorders. They can cause symptoms ranging from aggression, mania, and impulsiveness, to anhedonia, depression, and attention-deficits (Wessa et al., 2007; Menzies et al., 2008; Heller et al., 2009; Cubillo et al., 2010; Courtney et al., 2013; Wang et al., 2013; Salomons et al., 2014; Baggio et al., 2015).

In healthy subjects, acute and chronic stress can impair the structure and function of the entire fronto-striatal and fronto-limbic system (Arnsten, 2009). In this context, the amygdala is particularly important, because it is highly interconnected with these systems and is a key element in emotional processing and regulation. Functional connectivity between the amygdala and the PFC was found to be increased during emotional self-regulation tasks (Banks et al., 2007). Moreover, increased dopamine levels in the amygdala are associated with aggressiveness (Phelps and LeDoux, 2005).

Combining positron-emissions-tomography (PET) and transcranial magnetic stimulation (TMS), Strafella et al. (2001, 2003) were the first to demonstrate, that the functional modulation of the lateral frontal (Strafella et al., 2001) and the primary motor cortex (Strafella et al., 2003) *via* TMS has a significant effect on the dopamine release in the ipsilateral caudate (Strafella et al., 2001) and putamen (Strafella et al., 2003), as measured by a radiolabeled D2-receptor ligand. They used a repetitive TMS protocol of 10 Hz lasting 30 min and a stimulation intensity of 100% of the individual resting motor threshold (rMT).

TMS to the PFC is now increasingly used as a treatment tool for major depression and bipolar affective disorder (Johnson et al., 2013; Janicak and Dokucu, 2015). The rationale for using excitatory TMS to the left PFC is an imbalance in activity between the right and left PFC in these disorders. The lower activity of the left PFC can be enhanced using TMS. In these studies, stimulation duration is often very long, and stimulation intensity is set at or rather above of the individual rMT, which can induce high levels of pain or discomfort.

Intermittent theta burst transcranial magnetic stimulation (iTBS) has been shown to reliably increase regional excitability as well as functional connectivity between brain areas (Huang et al., 2005). Studies comparing more conventional TMS protocols with more recently introduced TBS protocols collectively found a comparable effectiveness in changing neuronal excitability (Zafar et al., 2008; Ziemann et al., 2008). Combining long-lasting effects on local and network activity with minimal stimulation time iTBS is a promising therapeutic tool for disorders of the fronto-striatal system (Brunelin et al., 2011; Li C.-T. et al., 2014; Bakker et al., 2015; Duprat et al., 2016). These studies typically apply iTBS to the PFC and measure a behavioral outcome variable through a standardized test. In a similar study on Parkinson patients (Benninger et al., 2011) found beneficial effects of iTBS on mood, but no improvement in other disease-related measures.

Nevertheless, the precise mechanisms of TMS-induced network modulations and the role of stimulation intensity has not been explored systematically and in detail yet. Therefore, we applied iTBS to the left PFC using sub and supra-resting-motor-threshold stimulation intensities to test parameters which combine strong network modulation and minimal side effects. The aim of this pilot study was to compare the effect of sub- vs. supra-threshold iTBS on functional connectivity of the entire fronto-striatal network by conducting resting state fMRI measurements before and after the iTBS. Additionally, we wanted to explore functional connectivity between the fronto-striatal network and the amygdala because of its importance for emotional-processing. Portions of the data published in this article have previously been presented as conference posters (Effnert et al., 2016a,b).

## Materials and Methods

### Participants

Our study was approved by the local ethical committee (protocol number: 003/15), and procedures involving human participants were in accordance with the ethical standards of the institutional and/or national research committee and with the 1964 Helsinki Declaration and its later amendments or comparable ethical standards. Informed consent was obtained from all individual participants included in the study.

Sixteen neurologically and mentally healthy, right handed (validated by the Edinburgh Handedness Inventory, Oldfield, 1971) participants were recruited (mean age = 27.63, SD = 6.95; 8 males). Participants were pre-screened for TMS and MRI exclusion criteria. Sensitivity to the TMS protocol was investigated prior to the experiment by applying the excitatory iTBS protocol to the dedicated prefrontal location and increasing the stimulation intensity stepwise to a maximum of 50% of the maximum stimulator output. Subjects could then decide whether or not the stimulation was too unpleasant and whether they still wanted to participate.

### Experimental Procedure

**Figure 1:**
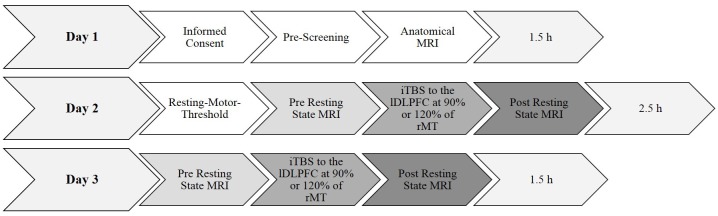
Summary of the experimental design and durations. Day 1 and day 2 took 1.5 h each. Determining the resting-motor threshold took approximately 45 min. All anatomical and functional magnetic resonance imaging (fMRI) scans took approximately 10 min each. The experimental procedure took in total approximately 5.5 h per participant. rMT, resting motor threshold.

A summary of the experimental procedure and the durations are shown in Figure 1. Each participant was invited into the laboratory on three separate days. The pre-selection procedure and informed consent were done on day 1. On day 2, the individual rMT was determined using a standardized protocol (Rossi et al., 2009; Rossini et al., 2015; see “Transcranial Magnetic Stimulation” section below). After a break of about 20 min, a 10 min baseline resting state MRI measurement was collected. For the following TMS measurement, the participant was brought outside the scanner room lying supine on the mobile scanner bed. The participants were registered with their anatomical data (see “Magnetic Resonance Imaging” section below for scanning parameters) and the iTBS lasting 3 min and 20 s was applied over the left PFC either at 90% or at 120% of their rMT. The order of the 90% and the 120% stimulation intensities was counterbalanced and alternated between subjects. After a 7-min-break the participants were rolled into the scanner again, lying in an unchanged position on the scanner bed and the 10-min-post resting state MRI measurement was conducted. During the scan participants were shown a small black fixation spot in the middle of a gray background. They were instructed to fixate the dot at all times, to relax, do not fall asleep, lie as still as possible, and to try not to think of anything in particular. The measurements on day 3 were conducted identically to day 2 by only varying stimulation intensity. The rMT was not determined again.

### Transcranial Magnetic Stimulation

To determine the individual rMT, an anatomical T1 MRI scan was used to locate the hand area of the left motor cortex. The subjects were placed in a comfortable chair and then their heads were registered to their individual anatomical T1 scan using neuronavigation software (TMS navigator, Localite GmbH, Sankt Augustin, Germany). The presumed hand area was identified visually through anatomical landmarks and stimulated with biphasic single pulses using a figure-of-eight coil (MagVenture C-B60) connected to a MagPro stimulator (X100 MagVenture, Farum, Denmark). Electrodes were fitted to the participant’s right index-finger and motor evoked potentials were monitored. Stimulation intensity was first increased in 2% steps until the hand area could be determined through a clear supra-threshold (>50 μV) motor evoked potential. Intensity was then reduced stepwise to find the lowest intensity to still induce a supra-threshold motor evoked potential.

The experimental excitatory iTBS (Huang et al., 2005) protocol consisted of 600 pulses spaced-out over 3 min and 20 s. It was comprised of 20 trains and 10 theta-bursts and was applied over the left DLPFC. Between each of the 2 s-long trains (50 Hz) there was an 8 s long pause. The stimulation site was determined by transforming the individual anatomical images into the Talairach system using the neuronavigation system (Localite TMS navigator) and marking the Talairach coordinates x/y/z = −45/45/35 as stimulation target. Additionally, individual anatomical landmarks (inferior and superior frontal sulcus) were taken into consideration to correct the location of the stimulation side in case the coordinate was outside of the DLPFC. This procedure was applied as suggested by Fitzgerald et al. (2009). They were able to demonstrate that the use of this neuro-navigational method to target a PFC site enhanced response to TMS treatment in depression, as compared to the formerly standard 5-cm procedure (locating the hand areal and simply measuring 5 cm in the sagittal plane on the scalp). The mean Talairach coordinates of the actual stimulation sites were x/y/z = −41/37/31 (*SD* = 5.33/14.8/9.83). The mean rMT was 43% (*SD* = 4.82) of the maximum stimulator output. The mean sub-threshold stimulation applied was 38% (*SD* = 3.91) and the mean supra-threshold was 50% (*SD* = 5.39) of the maximum stimulator output.

### Magnetic Resonance Imaging

MRI scans were measured on a Magnetom Prisma 3.0 T whole-body scanner (Siemens Medical Solutions, Erlangen, Germany). Anatomical data was acquired using a three-dimensional magnetization-prepared, rapid acquisition gradient-echo sequence (MPRAGE) with the following parameter: 300 repetitions, TR = 2,300 ms, TE = 2.98 ms, 9° flip angle, FOV = 256 mm, 176 sagittal slices, slice thickness = 1 mm and in-plane resolution = 1 × 1 × 1 mm.

Resting state MRI data were measured with a gradient echo planar imaging (EPI) sequence with the following parameters: TR = 2,000 ms, TE = 28 ms, 77° flip angle, FOV = 192 mm, 34 axial slices (interleaved acquisition), 3 mm slice thickness, EPI volumes and in-plane resolution = 3 × 3 × 3 mm. Each of the sequences lasted about 10 min.

### Pre-processing of Resting State Data and Functional Connectivity

MRI-data was analyzed using the Statistical Parametric Mapping software SPM10 (Welcome Department of Cognitive Neurosciences, London, UK) and Data Processing and Analysis for Resting-State Brain Imaging (DPABI, Yan et al., 2016) toolboxes running under Matlab R2012b (MathWorks Inc., Natick, MA, USA). Pre-processing of the rsfMRI data included the following steps: removal of first five volumes to discard saturation effects, slice time correction, realignment, segmentation, nuisance covariates regression with white matter and cerebrospinal fluid as regressors, head motion correction, head motion scrubbing as regressors, band pass filtering of the frequencies 0.01–0.08 Hz, spatial smoothing (5 mm FWHM) and detrending (removal of gradual shifts). The root-mean-square of the head motion translation parameters [displacement = square root (x^2^ + y^2^ + z^2^)] across all participants and sessions was 0.43 mm, with a session mean maximum of 1.44 mm and minimum of 0.15 mm (*SD* = 0.27 mm).

Seed-to-seed functional connectivity was calculated using the following 10 seed regions: individual stimulation site (sphere of 1 cm diameter, left DLPFC), right DLPFC, left and right caudate, left and right nucleus accumbens, left and right putamen, as well as left and right amygdala. For each region a left-side and a right-side seed were calculated individually. Three-dimensional seed masks were obtained from each participant’s individual T1-anatomy and then co-registered with the corresponding functional data. These masks were then used to extract a mean BOLD signal calculated from the time series of all mask voxels within the particular seed. Functional connectivity is thus the correlation between the mean BOLD signals of two areas. All correlation values are Fisher-Z-transformed. The resulting correlation maps were co-registered to the MNI standard template.

All further statistical processes were done using SPSS Statistics 23 (IBM Corp., Armonk, NY, USA). To address the question of whether the TMS stimulations modulated fronto-striatal connectivity, a mean BOLD signal was calculated for the entire striatum. This was done by averaging the mean signals of all above mentioned striatal seeds (caudate, nucleus accumbens and putamen). Fisher-Z-transformed correlations (i.e., functional connectivity) between this big striatal seed and the stimulation site seed (left DLPFC) were then calculated for each of the four measurements (90/120% × pre/post). This approach was chosen to avoid over-representation of the CA signals since this region comprises the biggest part of the striatum and thus the most voxels.

## Results

**Figure 2:**
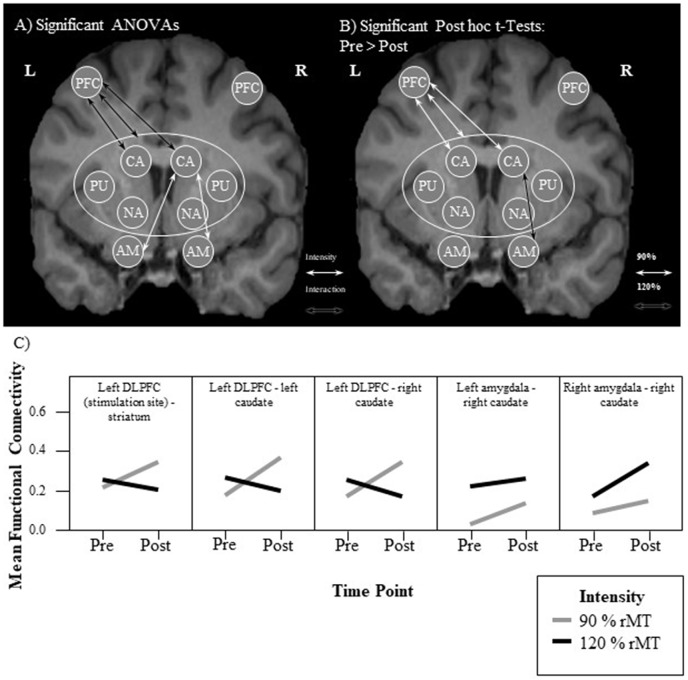
Visualization of results. **(A,B)** Each arrow represents a significant test result. Significant analysis of variance (ANOVA) effects are shown in **(A)** and significant paired *t*-tests in **(B)**. It can be seen that functional connectivity between the left prefrontal cortex (PFC; stimulation site) and the left and right caudate, respectively, as well as between the right caudate and the left and right amygdala, respectively, was modulated by the experimental stimulation. **(A)** White arrows indicate a significant intensity effect and black arrows a significant interaction effect. White ellipses indicate a seed region of interest. **(B)** Only pre-post transcranial magnetic stimulation (TMS) comparisons were calculated. All significant changes were increases from pre to post. Functional connectivity increased significantly only in the 90% condition between the stimulation site and the striatum. Analyzing the functional connectivity patterns between the stimulation site and each of the subregions of the striatum revealed that only the increase in functional connectivity between the stimulation site and the right and left caudate, respectively was significant. Additionally, functional connectivity between the right amygdala and the right caudate was increased only in the 120% condition. **(C)** Mean functional connectivity results. Shown are mean Fisher-Z-transformed correlations for the significant ANOVAs of functional connectivity between seeds, in the four different conditions 90% pre and post (gray line) and 120% pre and post (black line) measurements. Positive values indicate a positive coupling of signals between the two seeds. The first graph visualizes results between the stimulation site (left DLFC) and the entire striatum [consisting of left and right caudate (CA), left and right nucleus accumbens (NA), as well as left and right putamen (PU)]. AM, amygdala.

A 2 × 2 repeated measures analysis of variance (ANOVA) of the functional connectivity between the left DLPFC and the entire striatum revealed a significant interaction (*F*_(1,15)_ = 11.29, *p* = 0.004, $\eta_{\text{p}}^{2}$ = 0.43) between the two factors intensity (90% vs. 120%) and time point (PRE vs. POST). This effect size and observed power of 0.88 indicate a large effect (Cohen, 2013). a priori paired *t*-tests comparing the pre and post functional connectivity values for each intensity separately, revealed that the mean fronto-striatal functional connectivity was significantly increased after the 90%-stimulation (pre: *M* = 0.22, *SD* = 0.24; post: *M* = 0.35, *SD* = 0.22; *p* = 0.006), but not after the 120%-stimulation (pre: *M* = 0.25, *SD* = 0.26; post: *M* = 0.20, *SD* = 0.26;* p* = 0.27). Values are visualized in Figure 2 and summarized in Table 1. In both intensity conditions, functional connectivity was always positive and slightly decreases to a positive value closer to 0 in the 120% condition. Thus, the results demonstrate a decoupling of frontal and striatal activity, rather than negative coupling.

**Table 1:** Statistically significant seed-to-seed functional connectivity (FC) results.

Seeds/*Intensity*	Effect/*Post Hoc Pair*	SS	*df*	MS/MD	*F/t*	*p*	Adj. *p*	$\eta_{\text{p}}^{2}$	Obs. Power
lDLPFC—STR	Interaction	0.13	1	0.13	11.29	0.004	−	0.43	0.88
*90%*	*Pre-Post*	−	15	−0.13	−3.22	0.006	−	−	−
lCA—lDLPFC	Interaction	0.23	1	0.23	29.93	<0.001	0.003	0.67	0.999
*90%*	*Pre-Post*	−	15	−0.18	−4.15	0.001	−	−	−
rCA—lDLPFC	Interaction	0.26	1	0.26	14.87	0.002	0.035	0.50	0.95
*90%*	*Pre-Post*	−	15	−0.17	−3.13	0.007	−	−	−
lAM—rCA	Intensity	0.40	1	0.40	28.18	<0.001	0.005	0.65	0.999
rAM—rCA	Intensity	0.31	1	0.31	16.94	0.001	0.020	0.53	0.97
*120%*	*Pre-Post*	−	15	−0.17	−4.05	0.001	−	−	−

*Note. Results of significant ANOVAs and paired t-tests. Significant a priori t-test results are shown below each ANOVA row. Significance levels and observed power were computed using alpha = 0.05 (two-tailed). Negative t-values indicate an increase in functional connectivity and positive t-values a decrease from pre to post. lAM/rAM, left/right amygdala; lCA/rCA, left/right caudate; lDLPFC/rDLPFC, left/right prefrontal cortex; STR, whole striatum; SS, sum of squares; df, degrees of freedom; MS, mean squares; MD, mean difference; F, F-ratio; t, t-value; p, p-value; Adj. p, FDR (Benjamini and Hochberg) adjusted p-value, $\eta_{\text{p}}^{2}$, partial eta square; Obs. Power, observed power*.

To further explore the effect of the stimulation, a correlation matrix of the 10 seeds was created for each subject and each of the four conditions. These seed-to-seed-functional connectivity values were then subjected to repeated measures ANOVAs, resulting in 45 separate tests. The resulting multiple comparison problem was addressed by controlling the false discovery rate (FDR) using the Benjamini and Hochberg procedure (Benjamini and Hochberg, 1995). Planned *post hoc* paired *t*-tests were conducted to compare the pre vs. post iTBS measures only and *p*-values were adjusted using Bonferoni correction (*p* = 0.05/2). All significant results are summarized in Table 1, Figure 2.

This analysis revealed that the iTBS was able to modulate mainly fronto-caudal, as well as caudal-amygdala functional connectivity. Functional connectivity between the left-DLPFC and the bilateral caudal nuclei showed an interaction effect of the two factors time point of measurement and stimulation intensity, that matched the pattern of the whole-striatum analysis (see Figure 2C). This is functional connectivity increased from pre to post iTBS only in the 90% condition, but not in the 120% condition. Caudal-amygdala connectivity, on the other hand, showed a significant intensity effect only in the 120% condition, i.e., functional connectivity was higher in the 120% condition regardless of the time point of the measurement. *Post hoc* tests revealed a significant increase in functional connectivity between the pre and the post iTBS measurement for the right caudate and the right amygdala.

## Discussion

The aim of this study was to explore the effects of excitatory prefrontal iTBS at intensities below and above the rMT on the functional connectivity of the fronto-striatal brain network. Functional connectivity is a concept to quantify the synchronization i.e., correlation between brain areas as measured by correlation of BOLD signals. The frontal-cortical and striatal areas build a highly connected network that communicates through synchronization of neuronal activity. Low synchronization of activity and thus disrupted communication between those brain areas and desynchronization of activity can lead to aforementioned behavioral and emotional problems, but can also be induced externally to purposefully alter brain activity. This can, for example, be done by TMS.

The results of this study demonstrate the importance of choosing an optimal stimulation intensity when applying TMS in research or therapy. Differences in intensity led to significantly different functional connectivity patterns. The sub-threshold stimulation (90% rMT) was sufficiently high to directly strengthen connectivity of the left DLPFC and the caudate, which was not found in the 120% intensity condition.

Even though the exact mechanisms are still largely unclear (Ziemann and Siebner, 2015), it is empirically well established that certain TMS protocols can cause long-term-potentiation or depression-like plasticity (Ziemann et al., 2008). However, an increase in resting-state connectivity does not automatically indicate an increase in activation of one area, but rather an increase in BOLD pattern synchronization of connected areas. Nevertheless, several studies have found a direct linear relationship between the two measures (see for example Bohning et al., 2000; Nahas et al., 2001; Bestmann et al., 2005). Nahas et al., 2001, for example, delivered short 1 Hz trains of prefrontal TMS at 80%, 100% and 120% of the individual rMT, and found increased activation of the auditory cortex in all three conditions, contralateral prefrontal activity in the 100% condition and bilateral prefrontal activity in the 120% condition. Higher stimulation intensity was associated with higher BOLD signal under the coil and contra-laterally.

It is thus reasonable to interpret the changes in functional connectivity in the 90% condition in our study as a sign for increased network synchronization, caused by increased activity of the PFC. Nevertheless, the precise relationship between BOLD-signal changes and changes in functional connectivity is not straightforward and seems to rely highly on the involved areas and networks (Fox et al., 2012).

Interestingly, in the 120% condition, a very different pattern emerged. Bilateral amygdala activity was synchronized with the activity of the right caudate. The amygdala is not directly connected with the caudate (Robinson et al., 2012), thus caudate amygdala synchronization may be driven by shared regions, including those in the midbrain (Zhang et al., 2016) and the basal forebrain (Li C.-S. R. et al., 2014).

The lack of an increase of fronto-striatal functional connectivity in the 120% condition could possibly be explained by homeostatic mechanisms, that decrease activation and/or connectivity above a certain threshold (i.e., through surround inhibition). Stronger stimulation could induce stronger surround inhibition at the stimulation side in the PFC and connected areas. The mechanism of surround inhibition is well described in the sensory or the motor system, where it sharpens sensation or facilitates the selection of voluntary movements (Aungst et al., 2003; Sohn and Hallett, 2004; Angelucci et al., 2017).

Additionally, it is known that the state of the brain during TMS is crucial for the modulation effect. A similar, seemingly paradoxical TMS effect has, for example, been reported for the preconditioning with different transcranial direct current stimulation (tDCS) protocols. Lang et al. (2004) and Siebner et al. (2004), for example, applied excitatory and inhibitory tDCS to the motor cortex and found that the modulatory effect of a following TMS on the cortical excitability was unexpectedly increased through an inhibitory preconditioning and decreased for the excitatory tDCS. Interestingly, this pattern was not changed by the variation of the TMS protocol. Both inhibitory and excitatory TMS produced the same modulation pattern. One should, however, keep in mind that the cytoarchitecture of the PFC is vastly different to that of the primary motor and sensory cortices and it is unknown whether these phenomena can also be observed in the PFC. Thus, it is important to conduct more detailed research on the effects of magnetic stimulation on the PFC.

Even though the DLPFC was chosen as the target stimulation area in our study, because of its special role in cognition, other cortical areas such as the pre-supplementary motor areas show strong connections to the striatal structures as well (Zhang et al., 2011). It might thus be an equally good candidate as a stimulation site to enhance striatal activity. TMS is already used as a therapeutic tool. Its usefulness has mainly been proven for mood disorders, such as depression or bipolar disorder (see for example Johnson et al., 2013; Janicak and Dokucu, 2015). However, stimulation intensity and other protocol parameters are usually chosen rather randomly. These studies have typically used long continuous stimulation protocols of up to 20 min. Our study underlines the effectiveness of the comparatively short iTBS protocol of only 3.33 min, as previously shown by Huang et al. (2005). ITBS could potentially become even more beneficial for other disorders involving the dysfunction of the fronto-striatal system, such as Parkinson’s disease or prodromal Alzheimer dementia. The ultimate goal for research and clinical application should thus be to find reliable ways to individually determine the lowest possible and highest necessary stimulation intensity and the optimal protocol to induce desired changes in brain activity.

### Limitations

We did not systematically record perceived pain levels since participants were pre-screened to show a high tolerance against pain or discomfort during the TMS. This selection process could admittedly be prone to bias the sample. Inclusion of all participants and a systematic recording of subjective pain levels, on the other hand, would cause ethical and practical problems, and increase the issue of pain as a confounder. It has been reported that stress can impair the signaling pathway of the PFC (Arnsten, 2009). The influence of stress could indeed also explain the desynchronization of the fronto-striatal network in the 120% condition, as well as the increased amygdala activity. We did not implement a sham stimulation control group, because previous research in our lab has shown that participants can tell the difference between a sham and a real stimulation very well. We felt that the pre-measurement taken immediately before each of the stimulations is a better control measure since it allows controlling for short-term and long-term individual differences.

Nevertheless, whether it is homeostatic plasticity or the perception of pain and/or stress that causes the functional connectivity pattern should be addressed in further research by systematically recording subjective pain and stress levels during stimulation as well as pre-existing temporary brain states. Furthermore, since rMT is vastly variable inter- and intra-individually, and even a highly standardized procedure does not guarantee a reliable assessment, it is a problematic way of determining this stimulation parameter. Moreover, as we only used an intermittent theta burst protocol, it is unknown how stimulation protocol (e.g., low vs. high frequency, continuous vs. intermittent, etc.) and the current state of the network interact. Additionally, the number of subjects is rather low, which might weaken the conclusiveness of this pilot study.

Further research, incorporating a bigger sample size, different combinations of stimulation protocols (e.g., continues vs. intermittent theta burst) and intensities as well as sham stimulation, should be carried out to answer this question. Furthermore, studies comparing healthy participants and patients could further our understanding of the frontal-striatal network, its dysfunction and TMS as a possible treatment tool.

### Conclusions

Even considering its relatively small number of subjects, this pilot study demonstrates that small differences in iTBS intensity applied to the cortex can lead to vastly different effects in brain activity of a connected functional network. The fact that fronto-caudal functional connectivity was significantly more synchronized after sub-threshold stimulation of the PFC, when compared to after supra-threshold stimulation, reveals the existence of highly complex communication mechanisms based on activation synchronization of connected areas.

## Ethics Statement

This study was approved by and carried out in accordance with the recommendations of the ethical committee of the faculty of medicine of the RWTH Aachen University, Aachen, Germany (protocol number: 003/15). All subjects gave written informed consent in accordance with the Declaration of Helsinki.

## Author Contributions

Theoretical considerations, experimental design and experimental plan were prepared by FB, FM, and KS. The experimental setup and data collection was conducted by KS and IA. Data management and processing, statistical analysis and interpretation, as well as the manuscript preparation was carried out by IA.

## Conflict of Interest Statement

The authors declare that the research was conducted in the absence of any commercial or financial relationships that could be construed as a potential conflict of interest.

## Abbreviations

rsfMRI: Resting state functional magnetic resonance imaging
TMS: Transcranial magnetic stimulation
iTBS: Intermittent theta burst stimulation
ANOVA: Analysis of variance
rMT: Resting motor threshold
DLPFC: Dorso-lateral prefrontal cortex
BOLD: Blood oxygenation level dependent.
